# Zero-Shot Traffic Identification with Attribute and Graph-Based Representations for Edge Computing

**DOI:** 10.3390/s25020545

**Published:** 2025-01-18

**Authors:** Zikui Lu, Zixi Chang, Mingshu He, Luona Song

**Affiliations:** 1 School of Electronic Engineering, Beijing University of Posts and Telecommunications, Beijing 100876, China; 2 FedUni Information Engineering Institute, Hebei University of Science and Technology, Shijiazhuang 050018, China; 3 School of Cyberspace Security, Beijing University of Posts and Telecommunications, Beijing 100876, China; 4 School of Economics and Management, Beijing Information Science & Technology University, Beijing 100192, China; songlona@bistu.edu.cn

**Keywords:** edge computing, traffic classification, zero-shot learning, traffic representation, deep learning, graph neural networks

## Abstract

With the proliferation of mobile terminals and the rapid growth of network applications, fine-grained traffic identification has become increasingly challenging. Methods based on machine learning and deep learning have achieved remarkable results, but they heavily rely on the distribution of training data, which makes them ineffective in handling unseen samples. In this paper, we propose AG-ZSL, a zero-shot learning framework based on traffic behavior and attribute representations for general encrypted traffic classification. AG-ZSL primarily learns two mapping functions: one that captures traffic behavior embeddings from burst-based traffic interaction graphs, and the other that learns attribute embeddings from traffic attribute descriptions. Then, the framework minimizes the distance between these embeddings within the shared feature space. The gradient rejection algorithm and K-Nearest Neighbors are introduced to implement a two-stage method for general traffic classification. Experimental results on IoT datasets demonstrate that AG-ZSL achieves exceptional performance in classifying both known and unknown traffic, highlighting its potential for enhancing secure and efficient traffic management at the network edge.

## 1. Introduction

Traffic identification, critical for network management, plays a key role in improving the quality of service, preventing network intrusions and optimizing resource allocation [1,2,3,4]. In edge computing environments, where real-time processing and efficient resource management are essential, traffic classification faces unique challenges. The rapid emergence of privacy-enhancing technologies and protocols has further complicated this task [5]. Over 70% of network traffic now uses encryption algorithms to protect privacy [6], concealing the characteristics of traffic generated by malicious activities. Encrypted malicious traffic often appears statistically similar to benign traffic, making it difficult to classify using methods reliant on specific traffic patterns [7].

Furthermore, traditional traffic classification methods, primarily based on machine learning or deep learning, typically rely on supervised learning with pre-collected traffic data [8]. These approaches struggle to adapt to the dynamic and resource-constrained edge computing environments, as they cannot encompass all known traffic categories. Morever, with the proliferation of network protocols and services, the volume of unknown traffic categories continues to grow [5]. Consequently, there is an urgent need for innovative approaches to traffic identification [9,10].

To address this issue, it is crucial to consider the prediction of unknown traffic during classification, the task is commonly referred to as zero-shot learning (ZSL) [11]. One of the key concepts of ZSL is transfer learning [12], which involves adding an intermediate representation layer to bridge unknown and known classes, thereby leveraging knowledge from known classes to predict the categories of unseen samples.

Currently, research on zero-shot learning for encrypted traffic classification is relatively limited. Early studies primarily focused on the binary classification problem [9], dividing traffic into two categories: known traffic and unknown traffic. Although these methods consider the impact of unknown data, the practical value of binary classification remains controversial, as traffic classification requires finer-grained distinctions. Works [10,13,14] apply clustering algorithms to classify unknown traffic, but a common issue is the difficulty in determining the optimal number of clusters. Additionally, some researchers use reinforcement learning [15] and attention mechanisms [16] to identify unknown traffic. To address the data acquisition problem, studies [8] improve model performance by synthesizing unknown data. However, these methods lack interpretability.

Considering that ZSL often uses intermediate representations to connect unknown and known classes, a natural idea is to bridge them by studying shared characteristics. Notably, traffic is not naturally occurring, it is structured data designed by humans. Thus, traffic inherently contains common attributes that can be leveraged to transfer knowledge from seen classes to unseen classes. Specifically, the training data can be used to find unique patterns by learning relationships between datagrams and attributes, enabling fine-grained classification.

In this paper, we divide the task of identifying traffic into two stages: first, determining whether the network traffic belongs to unseen classes. If it is identified as belonging to the unknown category, we further map the traffic to an attribute label (the description of the attribute label is present in Section 4.1.2). Otherwise, we perform a fine-grained classification to identify the ground-truth label of the traffic. The gradient rejection strategy [10] and the KNN algorithm are introduced into the framework. The gradient rejection strategy is used to determine whether the traffic belongs to an unseen class, while KNN maps unknown traffic to attribute labels based on intermediate attribute representations. In summary, we propose a ZSL-based framework to solve the issue of identifying general encrypted traffic. The framework consists of two components: (1) Graph neural network (GNN) for traffic behavior embedding. Traffic bursts often reveal details of traffic interaction behavior [17]. Therefore, we first extract a traffic interaction graph based on bursts and then utilize GNN to extract features, generating the representation of traffic behavior. (2) Traffic attribute description. Attributes represent the most fundamental information of traffic and are not changed by environmental factors. Attributes cover various aspects of traffic, such as actions, transmission patterns, packet rates, and other characteristics. An attribute description is a sequence composed of multiple attributes of the flow, which is then embedded using simple recurrent units (SRU) [18] to generate an attribute representation.  The contributions of this paper are as follows:

- Conceptually, we propose a ZSL-based framework for general encrypted traffic classification that uses attribute representations as intermediates to address the challenge of predicting unknown categories. This framework enables knowledge transfer from known to unknown categories through attribute-based representations, effectively enhancing model generalization.
- Methodologically, the framework leverages traffic attribute representations to transform traditional classification labels into attribute labels that facilitate knowledge transfer. These attributes, which include basic traffic characteristics, are integrated with traffic behavior representations to enable robust classification of unknown categories.
- Innovatively, a novel burst-based traffic interaction graph is introduced to capture detailed traffic interaction features, where nodes represent datagrams and edges encode the interaction relationships between endpoints.
- Experimentally, extensive experiments on public datasets demonstrate that AG-ZSL achieves state-of-the-art performance in both fine-grained classification and zero-shot prediction.

The remainder of the paper is structured as follows: Section 2 reviews the related work, Section 3 outlines the preliminaries, Section 4 introduces the proposed framework, Section 5 presents the experiments and analysis, and Section 6 concludes the paper.

## 2. Related Work

### 2.1. Traffic Classification

Research on traffic identification has been widely applied across various domains and can be broadly divided into three categories: statistics-based, machine learning-based, and deep learning-based approaches. For discussion purposes, we summarize the related works in Table 1.

#### 2.1.1. Statistic Methods

The earliest traffic classification methods were based on specific attribute values and statistics, such as port-based techniques and deep packet inspection. However, these methods relied heavily on prior knowledge, and with the increasing use of dynamic allocation protocols, they have gradually become less effective.

#### 2.1.2. Machine Learning Methods

Machine learning methods primarily collected packet attribute values through side-channel information to classify traffic based on their states, which mitigates the impact of random content on accuracy. Taylor et al. [19] utilized packet sizes for training classifiers, while Al-Naami et al. [20] focused on temporal data. Panchenko et al. [21] used packet size and direction to classify traffic. These approaches rely heavily on expert knowledge and feature engineering, which lead to their ineffectiveness at classifying data with unknown labels.

#### 2.1.3. Deep Learning Methods

Wang [22] was the first to apply computer vision techniques to traffic classification by converting datagram bytes into grayscale images, enabling CNNs to classify encrypted traffic. Building on this approach, Bhat et al. designed Var-CNN [7], which accounts for the multidimensional characteristics of traffic by using convolutional kernels with various sizes to extract features. Additionally, natural language processing algorithms have also been widely applied to traffic classification tasks. Luo et al. [23] leveraged transformer models to capture semantic relationships for traffic classification. Huoh et al. [24] analyzed the relationships between traffic bytes and packet interactions for application classification. Mimetic-All [25] used burst traffic and communication protocols as contextual inputs, serving as an additional modality for application classification. He et al. [26] applied ALBERT to encrypted traffic classification, achieving an accuracy of 93.27%. However, since these methods did not fully consider the characteristics of datagram interactions, resulting in suboptimal model performance.

Compared to other models, GNNs are capable of handling non-Euclidean data, such as the relationships between packet length, and direction of transmission in flow. By mapping traffic into a graph structure, GNNs can capture features such as temporal and sequential relationships between datagrams. Additionally, GNNs handle variable-length traffic without the need for padding or truncation, as is required by CNNs or RNNs, thus avoiding the precision loss caused by padding or truncation. To fully utilize the meta information embedded in packet data and packet order, researchers have begun adopting GNNs for traffic identification. Huoh et al. [27] were the first to convert encrypted traffic into a graph structure, preserving its temporal and structural information as well as overcoming the limitations of fixed input shapes required by traditional methods. This approach significantly improved classification accuracy. Shen et al. [28] captured the multidimensional interaction features between clients and servers through graph structures, transforming the DApp traffic classification problem into a graph classification task. Yang et al. [29] transformed the traffic sessions into graphs and proposed a semi-supervised classifier. Pang et al. [30] proposed a chained graph model to maintain the chained compositional sequence of the datagrams. Compared to previous methods, our approach emphasizes burst characteristics in the graph structure design, which more effectively highlights traffic interaction features.

### 2.2. Zero-Shot Learning

Zero-shot learning addresses the challenge of classifying unseen classes without labeled data by learning transferable semantic representations from known to unknown classes. In contrast, traditional deep learning-based traffic classification models are trained in a supervised manner using closed-world data, with performance typically reliant on class balance and the amount of labeled data available in the dataset [12,31].

Current research has explored the use of ZSL for classifying unknown encrypted traffic. Payap et al. [32] applied triplet networks in traffic classification, using contrastive learning to optimize the loss function and address challenges caused by network condition variations and data distribution inconsistencies. Hu et al. [8] proposed a framework that leveraged attributes from known classes and GAN-generated data to enhance the classification capability for unknown classes. Yang et al. [9] developed the GradBP algorithm, which detected novel and unseen traffic patterns using gradient backpropagation. Zhang et al. [10] introduced the HyperVision system, which utilized a flow interaction graph to detect unknown encrypted traffic. Wu et al. [16] proposed a model based on the self-attention mechanism to classify known and unknown traffic. Fu et al. [13] and Zhao et al. [14] both classified unknown traffic through clustering. In comparison to these methods, AG-ZSL connects known and unknown classes by intermediate semantic representations, and the use of the gradient rejection strategy can further enhance the interpretability of the approach.

## 3. Preliminary

In real-world scenarios, unseen traffic primarily originates from two sources. First, new applications or protocols generate unknown traffic, and second, traffic undergoes “morphing” after encryption, causing significant differences in feature distribution compared to training data, which increases the difficulty of traffic classification. However, some traffic features exhibit similarities during transmission, meaning that the characteristics of unknown traffic may partially overlap with those of known traffic. This provides a theoretical basis for knowledge transfer in zero-shot classification.

Compared to other methods, AG-ZSL additionally defines traffic attribute labels, which are attribute sets consisting of multiple attribute values that serve as transferable knowledge for unknown traffic classification. Network traffic needs to be fully represented, for which a burst-based traffic interaction graph is designed. The graph helps capture the temporal and spatial information within sessions and is learned by a GNN to generate the graph vector. Then, SRU generates the attribute vector based on the attribute labels of known traffic. In the model training phase, the objective of AG-ZSL is to improve the similarity between the traffic graph vector and its corresponding attribute vector. To avoid the impact of inconsistencies in data distribution on the training objective, we use the Euclidean distance [11] to measure the similarity between the two vectors. Below, we provide detailed explanations of the traffic interaction graph, attribute description, and objective of the framework.

### 3.1. Traffic Interaction Graph

The burst-based traffic interaction graph is used for a fine-grained representation of traffic. A burst is a collection of adjacent packets within a flow that have the same direction. From the application layer perspective, bursts reflect traffic characteristics and effectively illustrate traffic transmission patterns. Typically, when two endpoints communicate, the complete content is transmitted in the form of bursts. Therefore, bursts can capture the structural information and sequential relationships associated with traffic categories.

**Figure 1:**
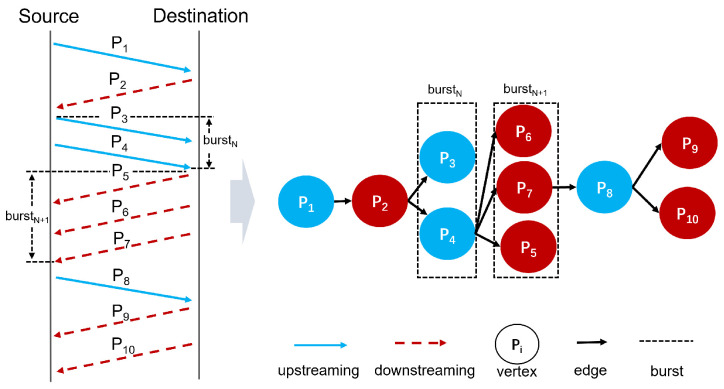
Traffic interaction graph based on burst.

We design a traffic interaction graph based on bursts, as shown in Figure 1. In this graph, each node represents a packet, denoted as $p_{1\cdots k}$. The value of each node corresponds to the length of the associated packet. Upstream packets and downstream packets are marked in blue and red, respectively. Each edge represents the relationship between two consecutive bursts. Importantly, each packet is connected with the last packet of the previous burst. For instance, in Figure 1, the last packet of $burst_{N}$ is $p_{4}$, which is connected to every packet in $burst_{N+1}$, as well as the dash lines indicate the boundaries of bursts. Additionally, the directions of the arrows indicate the chronological relationship between packets.

### 3.2. Attribute Description

Traditional methods usually focus on single tasks, such as classifying network types, applications, or anonymity, with each task relying on some attributes. Since traffic is human-defined data, typically generated by terminal activities and transmitted over the network only when protocol requirements are met, the same category often exhibits similar patterns. For example, datasets used for service classification can also be applied in application identification. By combining attributes from multiple tasks into a unified attribute description, the commonalities among these traffic can be better revealed and leveraged for more fine-grained traffic classifications.

To describe traffic more accurately, it is essential to analyze the factors that may affect datagrams during flow generation and transmission. Referring to the work [8], traffic actions and protocols are crucial for traffic transmission patterns. Therefore, we consider action and protocol as two factors for the traffic attribute description. In addition, through the analysis of existing data, we find significant differences between normal and malicious traffic in terms of transmission mode and frequency (the analysis of transmission mode and frequency is presented in Section 5.2.3). Hence, in this framework, we define the traffic attribute description to include five factors: action, mode, type, protocol, and frequency. Among these, action refers to the behavior generating the traffic, mode refers to the transmission pattern of the traffic, type indicates the traffic is normal or anomalous, protocol refers to the type of protocol applied in the traffic, and frequency refers to the number of packets transmitted per unit time. The corresponding attribute values for each factor are shown in Table 2.

Among these attributes, action, protocol, and type are objective attributes that can be directly labeled. However, mode and frequency require further data statistics and analysis. The attribute values for mode include burst, spike, and steady. Here, burst indicates a high proportion of bursts within a flow, spike represents noticeable peaks in traffic transmission, and steady denotes a generally stable flow during transmission. The thresholds for spike and steady are determined by calculating the Coefficient of Variation (CV).

(1) CV=σ/μ

Here, σ represents the standard deviation of all packets within a flow, and μ represents the average packet size within the flow. frequency refers to the number of packets transmitted per unit time in a flow. The packet rate is defined as the number of packets per second. frequency includes two attribute values: fast and slow. In Section 5.2.3, we will analyze the threshold corresponding to the attribute value of mode and frequency.

### 3.3. Goal

Let $L_{s}$ = $\left\{l_{s1},l_{s2},\cdots ,l_{sn}\right\}$ represent known labels, and $L_{un}=\left\{l_{un1},l_{un2},\cdots ,l_{unm}\right\}$ represent unknown labels. Similarly, $T_{s}=\left\{t_{s1},t_{s2},\cdots ,t_{sn}\right\}$ represents known traffic, and $T_{un}=\left\{t_{un1},t_{un2},\cdots ,t_{unm}\right\}$ represents unknown traffic. The attribute label is defined as A = $\left\{a_{1},a_{2},\cdots \right\}$, where $a_{i}=\left\{v\left(action\right),v\left(mode\right),v\left(type\right),v\left(protocol\right),v\left(frequency\right)\right\}$ with $a_{i}\in R$. v(·) represents the specific value of the attribute. Assuming the training set contains *N* samples, each sample consists of seen traffic $t_{si}$, ground-truth label $l_{si}$, and the attribute label $a_{i}$, denoted as $E_{i}={\left\{t_{si},l_{si},a_{i}\right\}}_{i=1}^{N}$.

The goal of AG-ZSL is to train two models: the traffic behavior representation model *F* and the attribute representation model *M*, with the training objectives outlined as follows:

(2) $$F\left(t_{si}\right)\to l_{si}$$

(3) $$dis(F\left(t_{si}\right),M\left(a_{i}\right))\to 0$$

where dis denotes the Euclidean distance. $F(t_{si})$ represents the known traffic behavior embedding, and $M(a_{i})$ represents the attribute embedding. The two models are trained by minimizing the distance between their generated embeddings, ensuring semantic alignment between the input traffic and the corresponding attribute description based on the training set. This approach enables $F(t_{si})$ to perform fine-grained classification. Additionally, when encountering unknown classes, *F* and *M* can be used to predict attribute labels for samples, providing a basis for the subsequent analysis of unknown traffic.

**Figure 2:**
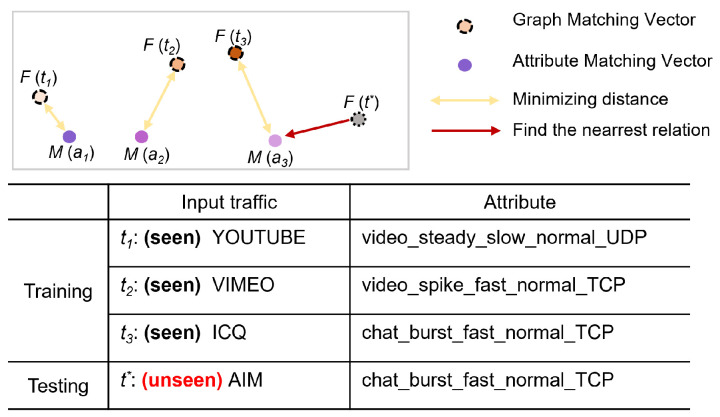
The training and testing objectives of AG-ZSL.

The inference phase includes two objectives: first, to determine whether the traffic is unknown, and second, to perform fine-grained classification by identifying the ground-truth label of known traffic or mapping unknown traffic to attribute labels. Figure 2 illustrates an example of AG-ZSL predicting unknown traffic, where $t_{1}$, $t_{2}$, $t_{3}$ represent YOUTUBE, VIMEO, and ICQ traffic, respectively, and $a_{1}$, $a_{2}$, $a_{3}$ are their corresponding attributes sets, as well as $t^{*}$ represents unknown traffic. The models *F* and *M* have been trained, and the attribute representations $M(a_{1})$, $M(a_{2})$ and $M(a_{3})$ are saved. Using KNN, the attribute representation that is close to $F(t^{*})$ can be found, with the corresponding attribute description serving as the attribute label for the unknown class.

## 4. The Proposed Framework

**Figure 3:**
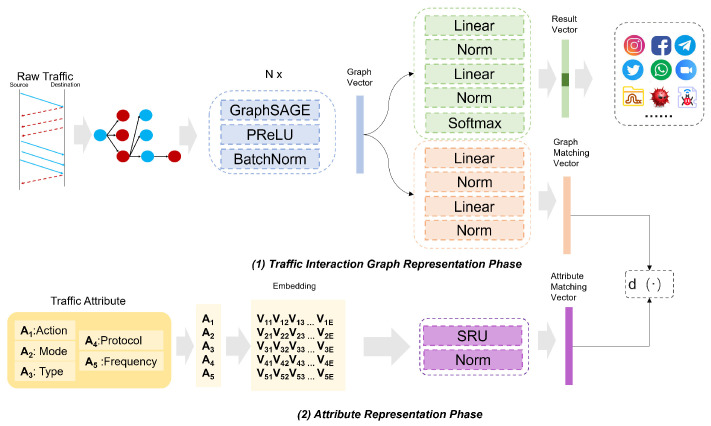
AG-ZSL training phase.

**Figure 4:**
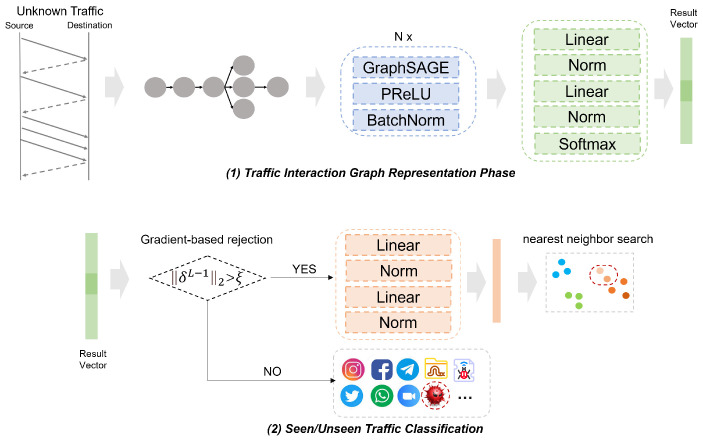
AG-ZSL inference phase.

The AG-ZSL architecture is illustrated in Figure 3 and Figure 4. The framework primarily consists of GNN and SRU, and it includes two stages: the model training phase and the zero-shot prediction phase. We train the framework under a multi-task method, with one task focusing on classifying the ground-truth labels of traffic, and the other task aiming to minimize the distance between traffic behavior representations and attribute representations. In the inference phase, AG-ZSL predicts either the ground-truth label or the attribute label for new traffic.

### 4.1. Model Training

The objective of the training phase is to generate two types of vectors: the result vector, which can be utilized for fine-grained classification based on traffic behavior, and the graph matching vector, which is designed to align with attribute representations. These two representations are based on the graph vector and are formed by different linear layers. As illustrated in Figure 3, the training phase is divided into two parts: traffic interaction graph representation and attribute representation.

#### 4.1.1. Traffic Behavior Representation

AG-ZSL employs GraphSAGE for the fine-grained representation of network traffic. GraphSAGE is an inductive learning model based on graph-structured data, and its core idea is to generate representations for target nodes by aggregating the features of neighboring nodes [33]. Aggregation functions are used to combine the features of these neighboring nodes with the features of target nodes to generate new feature representations. Based on this method, AG-ZSL can better learn the representation of burst traffic from adjacent neighbors. When it comes to training, the first step is to construct a burst-based traffic interaction graph from the raw traffic to serve as the input to the model. The packets are divided into different flows based on the five-tuple (source IP, destination IP, source port, destination port, protocol). Each flow $t_{i}$ is then transformed into a burst-based traffic interaction graph using the method mentioned in Section 3.1, denoted as $t_{i}\to G\left(t_{i}\right)$, where G() represents the process of generating the traffic interaction graph. This graph $G(t_{i})$ is then fed into a multi-layer GraphSAGE model. At each layer, GraphSAGE aggregates information from the current node and its neighbors to generate new node embeddings.

Following each GraphSAGE layer, the PReLU activation function is applied to introduce non-linearity into the network. The advantage of using PReLU is that when the output of GraphSAGE is negative, the activation function’s output is not always zero, which can help alleviate the vanishing gradient problem [34]. In addition, batch normalization is used to ensure that the input distribution remains consistent, thereby stabilizing the learning process. The readout function is employed to aggregate the features of all nodes, producing a global graph embedding vector.

The graph embedding vector is then fed into a network $f_{c}$, which consists of linear layers and normalization layers, to generate the result vector. This result vector is subsequently passed through the softmax function to predict the ground-truth label. In parallel, we design an additional linear network $f_{m}$, which generates a graph-matching vector. This vector will be utilized in the subsequent attribute-matching phase.

#### 4.1.2. Attribute Representation

Each traffic, in addition to the ground-truth label, also has an attribute label, which is an attribute description composed of five attribute values. First, all the attribute values that appear during the training phase are collected and a dictionary is constructed based on these values. Then, the attribute labels are encoded into vectors using the dictionary. These vectors serve as the input to the SRU and finally output the attribute matching vector.

The training phase consists of two objectives: the first is to maximize the classification accuracy based on the result vector using cross-entropy loss, and the second is to minimize the distance between the input traffic and its attribute description while maximizing the distance between the input traffic and other attribute descriptions.

(4) $$L_{c}=-\frac{1}{N}\sum_{N}P(y_{pred}^{i}|\theta )$$

(5) $$L_{d}=-\frac{1}{N}\sum_{N}\left[\max\left(0,\gamma +dis\left(f_{m}\left(f_{g}\left(t_{uni}|\theta \right)\right),m\left(a_{i}\right)\right)-\min_{j\neq i}dis\left(f_{m}\left(f_{g}\left(t_{uni}|\theta \right)\right),m\left(a_{j}\right)\right)\right)\right]$$

(6) $$L=\alpha L_{c}+\left(1-\alpha \right)L_{d}$$

where $L_{c}$ represents the cross-entropy loss, $L_{d}$ represents the distance loss between the traffic representation and its attribute representation, *i* and *j* represent different sample indices, $y_{pred}$ denotes the prediction vector based on the graph vector, *P* stands for the probability function, θ represents the model parameters, γ indicates the margin for distance and α denotes the loss weighting factor, and $f_{g}$ represents the function that generate graph vector.

### 4.2. Zero-Shot Prediction

As illustrated in Figure 4, the zero-shot prediction phase begins by formalizing the test data as $G(t_{uni})$, which serves as the input to the model. This prediction phase consists of two parts. First, the framework determines whether the traffic is unknown. AG-ZSL employs a gradient rejection strategy to identify whether the traffic belongs to an unknown class. The process of the gradient rejection strategy is as in Algorithm 1. This strategy leverages backpropagation gradients, using “shadow training” during inference to compute the gradient of the current classification result for the penultimate layer’s weights. This reflects the model’s sensitivity near the decision boundary. The gradient corresponds to input data near the decision boundary, indicating a possible unknown category. The computation [9] of gradient rejection is given as follows:

(7) $$\delta^{I}=\nabla L⊙\sigma^{\prime }\left(z^{I}\right)$$

(8) $$\delta^{I-1}=\left(W^{I^{T}}\delta^{I}\right)⊙\sigma^{\prime }\left(z^{I-1}\right)$$

(9) $${∥\delta^{I-1}∥}_{2}>\xi$$

where $\delta^{I}$ and $\delta^{I-1}$ represent the gradients of the last layer and the penultimate layer of neural network *z*, ∇L represents the partial derivative, $\sigma^{\prime }\left(z^{I}\right)$ and $\sigma^{\prime }\left(z^{I-1}\right)$ are the derivatives of the activation function with respect to the output of layer *I* and layer I−1, ξ denotes the threshold for determining whether the traffic is unseen, and $W^{I-1}$ is the weight between layer I−1 and layer *I*. If ${∥\delta^{I-1}∥}_{2}$ > ξ, the traffic is classified as an unknown class. In this case, $f_{m}$ is applied to represent $f_{g}\left(t_{uni}\right)$, generating the graph matching vector. KNN is then performed on this vector to find the closest attribute representation vector $M(a^{*})$. We then infer that the attribute label of $t_{uni}$ is $a^{*}$.
**Algorithm 1:** Gradient Rejection Strategy
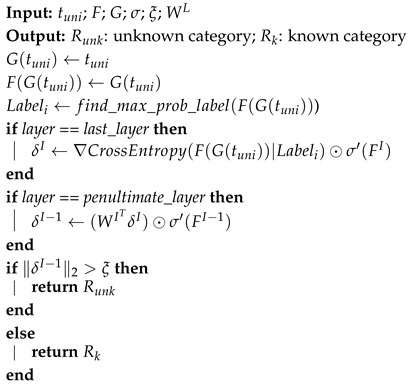


## 5. Experiment

In this section, we compare AG-ZSL with existing methods to evaluate the performance of the proposed framework in both fine-grained classification and unknown class prediction.

### 5.1. Dataset

We evaluate the framework on two public datasets: ISCX-VPN [35] and ToN-IoT [36]. ISCX-VPN includes popular VPN protocols such as OpenVPN and OpenConnect. Considering the encapsulation and service types of the traffic, this dataset contains traffic from 12 different applications. ToN-IoT is collected from a realistic, large-scale network designed at the Cyber Range and IoT Labs of SEIT and UNSW, which includes nine types of malicious traffic. Unlike other studies, in addition to the ground-truth labels, we further mark each traffic with an attribute label. The ground-truth labels and attribute labels for these two datasets are shown in Table 2.

Since the ISCX-VPN dataset is imbalanced, we sample 5000 packets for each application, resulting in a total of 60,471 packets across 4753 flows. The sampled dataset of ISCX-VPN is described as $D_{1}$. Similarly, for the ToN-IoT dataset, we sample 4000 packets for each type of malicious traffic, resulting in a total of 36,249 packets across 3326 flows, which is described as $D_{2}$. Details of these datasets are provided in the Table 3.

To demonstrate the robustness of the proposed method, the experiments include fine-grained classification and zero-shot prediction, which are described as $Exp_{1}$ and $Exp_{2}$, respectively. The attribute labels included in $D_{1}$ and $D_{2}$ can be found in the Appendix A.

### 5.2. Implementation

#### 5.2.1. Hyperparamters Setting

A key step in model training is determining the hyperparameters to balance model performance between underfitting and overfitting. To efficiently find suitable hyperparameters, the proposed framework utilizes interval search to identify the optimal combination. The implementation details of AG-ZSL are summarized in Table 4.

#### 5.2.2. Experiment Setting

All experiments are conducted on a Ubuntu system using PyTorch 1.8.0 with an NVIDIA RTX 3090 (NVIDIA, Santa Clara, CA, USA). For both $D_{1}$ and $D_{2}$, 70% of the data is used for training, 10% for validation, and 20% for testing. All experiments are performed in a controlled environment, and a ten-fold cross-validation method is used to evaluate the framework’s performance.

We evaluate and compare the performance of the methods using four typical metrics, including accuracy (ACC), precision (PRE), recall (REC), and F1 score, and the calculation methods of these metrics are as follows:

(10) $$ACC=\frac{TP+TN}{TP+TN+FP+FN}$$

(11) $$PRE=\frac{TP}{TP+FP}$$

(12) $$REC=\frac{TP}{TP+FN}$$

(13) $$F1=2*\frac{PRE*REC}{PRE+REC}$$

#### 5.2.3. Attribute Label Threshold Setting

Since the attribute values for mode and frequency need to be calculated based on statistical analysis, it is necessary to obtain the cumulative distribution function (CDF) of burst ratio, coefficient of variation, and packet rate in $D_{1}$ and $D_{2}$.

**Figure 5:**
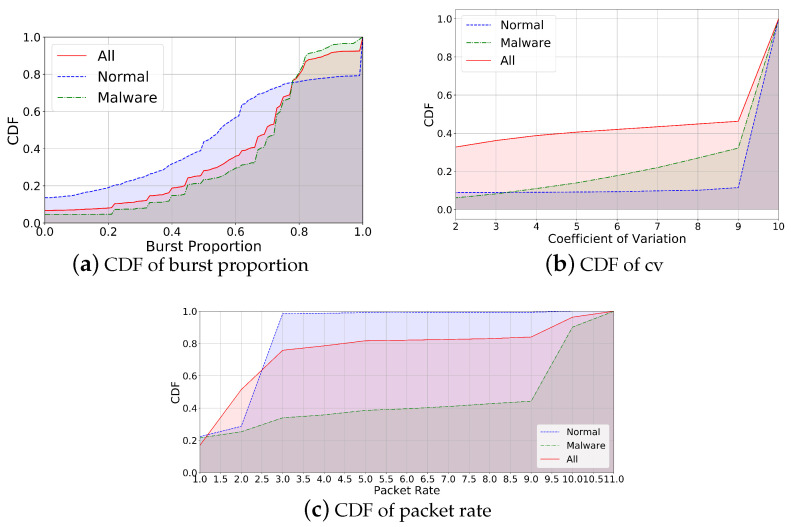
Data statistics: CDF of burst proportion, cv, and packet rate.

Based on the statistical results, we further determine the threshold corresponding to the attribute values of mode and frequency. Figure 5a shows the proportion of bursts in normal, malicious, and all traffic. From Figure 5a, we can see that 60% of normal traffic has a burst proportion of 61%, while only 30% of malicious traffic meets this condition. Figure 5b shows the proportion of coefficient of variation in normal, malicious, and all traffic. We can learn that 30% of malicious traffic has a CV between 2 and 9, while over 90% of normal flows have a greater CV than 9. Figure 5c shows the proportion of packet rates in normal, malicious, and all traffic, which demonstrates that over 95% packet rates of normal traffic are 3, while only 30% of malicious flows have a packet rate of 3.

Through the statistical analysis of normal and malicious traffic, we define the values with significant differences between normal and malicious flows as threshold attribute values. The specific threshold values are shown in the Table 4.

### 5.3. Experiments Results

We evaluate the performance of fine-grained classification and zero-shot prediction for AG-ZSL, using FS-NET [37] and TF [32] as the baseline comparison methods.

#### 5.3.1. Fine-Grained Classification Experiments

**Figure 6:**
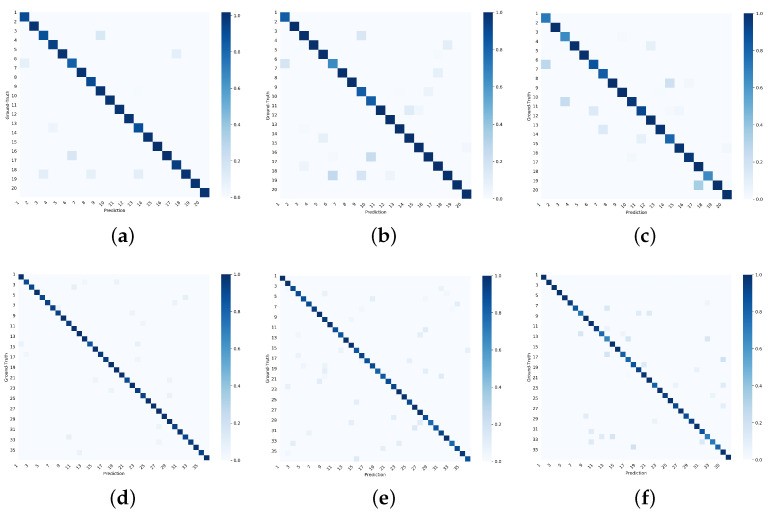
Confusion matrix on $D_{1}$ and $D_{2}$. (**a**) Confusion matrix of accuracy for AG-ZSL on $D_{1}$. (**b**) Confusion matrix of accuracy for TF on $D_{1}$. (**c**) Confusion matrix of accuracy for FS-Net on $D_{1}$. (**d**) Confusion matrix of accuracy for AG-ZSL on $D_{2}$. (**e**) Confusion matrix of accuracy for TF on $D_{2}$. (**f**) Confusion matrix of accuracy for FS-Net on $D_{2}$.

The classification results of the ground-truth label for $D_{1}$ and $D_{2}$ are shown in Table 5 while the attribute label results are shown in Figure 6. As shown in Table 5, our method significantly outperforms the baseline methods FS-Net and TF across all metrics. In $D_{1}$, the proposed framework achieves an accuracy of 0.9571 and a recall of 0.9613, while FS-Net and TF report accuracies of 0.9133 and 0.9171, and recalls of 0.9254 and 0.9257, respectively. AG-ZSL also demonstrates strong performance in terms of precision and F1 score, highlighting the clear advantage in fine-grained identification of encapsulated traffic. Similarly, the results in $D_{2}$ are outstanding, with the proposed framework achieving an accuracy of 0.9659 and a recall of 0.9659, compared to FS-Net’s and TF’s accuracies of 0.9146 and 0.9213, indicating that our method consistently delivers superior classification performance across various scenarios.

Figure 6 shows the confusion matrix of accuracy for AG-ZSL, TF, and FS-Net on the $D_{1}$ and $D_{2}$. The x-axis represents the predicted categories by the model, while the y-axis represents the attribute labels. Given the large number of labels involved in the experiments, the categories are represented by numerical labels, and the corresponding attribute labels can be found in Appendix A. The diagonal represents the ratio of the number of predicted samples to the actual number of samples, with darker colors indicating better performance.

In these experiments, AG-ZSL exhibits a high classification accuracy across most categories. In contrast, TF and FS-Net show more misclassification issues in certain categories, particularly with high similarity between classes. Our method, however, demonstrates a stronger ability to distinguish between different traffic classes, especially under complex traffic patterns, further emphasizing its superior performance.

#### 5.3.2. Zero-Shot Prediction Experiments

In $Exp_{2}$, if the traffic to be classified belongs to a known category, it is the model inference ground-truth label of the traffic. Otherwise, the model predicts the attribute label. In this experiment, 12 categories from $D_{1}$ and 20 categories from $D_{2}$ are sampled to evaluate the model’s performance. From $D_{1}$, four categories are selected as unknown classes, while from $D_{2}$, six categories are selected as unknown classes. The specific unknown categories are detailed in the Appendix A.

**Figure 7:**
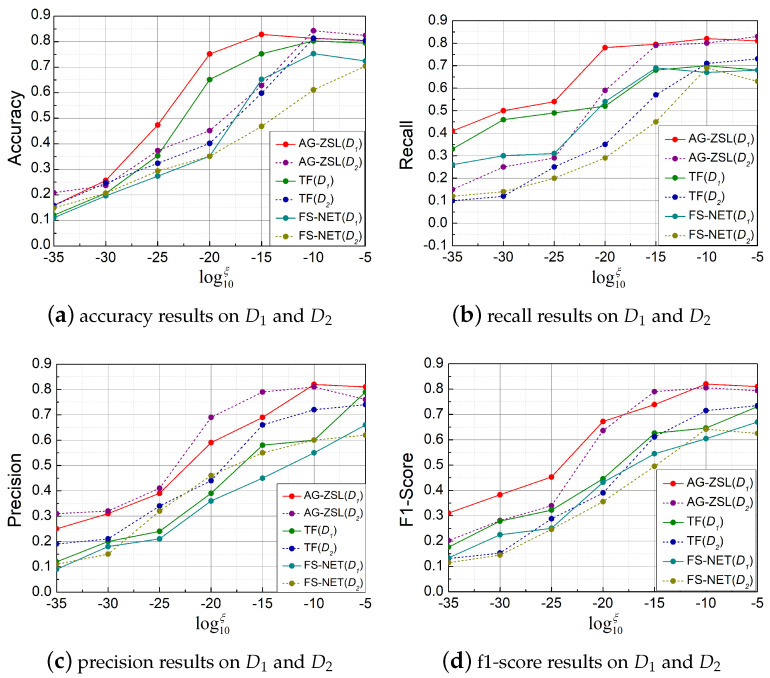
Comparison of the results of different methods under various ξ.

Figure 7 illustrates the results of three methods on unseen classes. By adjusting ξ, we can evaluate the model’s performance under different parameters. Figure 7a presents the accuracy in the $D_{1}$ and $D_{2}$. Our method significantly outperforms the baseline methods, especially when $log^{\xi }$ is low (between −35 and −20). In $D_{1}$, AG-ZSL shows a clear advantage, reaching around 0.84 when $log^{\xi }$ equals −15, while the accuracy of TF and FS-Net remains below 0.75. Figure 7c shows precision, where our method maintains superior performance, especially in the low $log^{\xi }$ range (−35 to −25).

Table 6 shows the performance of three methods in zero-shot prediction on $D_{1}$. The last four classes represent the unknown classes. It can be seen that AG-ZSL achieves the best results in the prediction of all 12 classes. When only looking at the prediction results of the unknown classes, our method has a maximum improvement of 12% in accuracy.

#### 5.3.3. Evaluation of Complexity

In this section, we will analyze the complexity of AG-ZSL, including the trainable parameters and time cost. As shown in Table 7, we present the model size, training time, and inference time. The training time refers to the phase where the model minimizes the Euclidean distance between the traffic behavior representation and the attribute representation. The inference phase includes generating the result vector, executing the gradient rejection strategy, and performing the nearest neighbor search. We define the time consumption of the proposed method as 1, serving as the baseline for the time cost of other methods.

From Table 7, it is shown that our model occupies a larger space, as our approach integrates multiple models, leading to more parameters. In terms of time cost, in the training phase, AG-ZSL takes significantly longer than the other two methods, as it requires generating two representations for a single sample and optimizing the distance between them. However, in both fine-grained classification and zero-shot prediction tasks, our method outperforms others, with a maximum recall rate improvement of 10%.

## 6. Conclusions

In this paper, we propose AG-ZSL, a zero-shot learning framework designed for the classification of both known and unknown traffic. This framework effectively addresses the challenge of identifying unseen traffic for edge nodes by leveraging both graph and attribute representations. The combination of traffic interaction representation and attribute representation enables the approach to classify both known traffic and infer the attributes of unknown traffic. To further enhance the generalization of the model, we introduce a gradient-based rejection strategy and a KNN algorithm.

To further validate the effectiveness of AG-ZSL, we plan to expand our experimental scope to include a broader range of datasets, encompassing more diverse types of network traffic. Morever, by collecting datasets that capture new traffic patterns and protocols, we can better evaluate the model’s generalization capabilities. In addition, we will focus on the impact of data drift on the model.

## Figures and Tables

**Table 1:** Related works in encrypted traffic identification.

Category	Method	Classifier	Raw Data	Burst	Sequential Information	Zero-Shot Prediction
Statistic method	DPI	-	-	-	-	-
Machine learning method	CUMUL	SVM	-	-	-	-
	AppScanner	Random Forest, SVM	-	-	-	-
	BIND	SVM, Random Forest	-	√	-	-
Deep learning mehtod	CNN	CNN	√	-	-	-
	Var-CNN	CNN	√	-	-	-
	FS-Net	BiGRU	√	-	-	-
	MIMETIC-ALL	CNN + BiGRU	√	√	-	-
	TF	Triplet network	√	-	√	√
	Geometric Learnng Model	GNN	√	√	√	-
	GraphDApp	GNN	√	√	√	-
	Flow-Based GNN	GNN	√	-	√	-
	ZSL framework	FAE-G + SRU	√	-	√	√
	AG-ZSL	GNN + SRU	√	√	√	√

**Table 2:** Ground-truth label and attribute labels used in datasets.

Dataset	Ground Truth	Attibute Label				
		Action	Mode	Type	Protocol	Frequency
ISCX-VPN	facebook, aim, skype, gmail, youtube, icq, vimeo, spotify, twitter, netflix, hangouts, torrent	vedio, audio, file, chat, emaiil	burst, spike, steady	normal	tcp, udp, http, ssh, ntp, dns, pop3, imap	fast, slow
ToN-IoT	scanning, XSS, DDoS, Dos, MITM, injection, backdoor, runsomware, password	polling, injection, control	burst, spike, steady	malware	tcp, udp, http, dns, ntp, ssh, pop3, imap, ftp	fast, slow

**Table 3:** The detail of datasets used in experiments.

Dataset	# Flow	# Packet	# Ground-Truth Label	# Attribute Label
$D_{1}$	4753	60,471	12	20
$D_{2}$	3326	36,249	9	36

**Table 4:** Hypberparamters for AG-ZSL.

Hyperparamters	Search Range	Final
Depth of GraphSAGE	[2, 3, 4, 5]	3
Embedding Size of GraphSAGE	[64, 128, 256]	256
Number of Neighbors	[5, 10, 15, 20]	5
Learning rage	[0.0001, …, 0.005]	0.001
Hidden Unit Size of SRU	[128, 256, 512]	512
Dropout	[0, …, 0.5]	0.1
Activation Functions	[Tanh, PReLu, Relu]	PReLu
Batch Size	[30, …, 300]	64
Optimizer	[Adam, RMSProp, SGD]	Adam
The threshold for the burst mode	[0, …, 1.0]	>0.6
The threshold for the spike mode	[0, …, 1.0]	>0.9
The threshold for the steady mode	[0, …, 1.0]	<=0.9
The threshold for the fast frequency	[0, …, 10]	>3
The threshold for the slow frequency	[0, …, 1.0]	<=3

**Table 5:** Comparison results on $D_{1}$ and $D_{2}$.

Dataset	Method	Accuracy	Recall	Precision	F1
$D_{1}$	TF	0.9171	0.9257	0.9182	0.9220
	FS-Net	0.9133	0.9254	0.9168	0.9211
	AG-ZSL	0.9571	0.9613	0.9578	0.9596
$D_{2}$	TF	0.9213	0.9213	0.9268	0.92412
	FS-Net	0.9146	0.9146	0.9201	0.9174
	AG-ZSL	0.9659	0.9659	0.9676	0.9667

**Table 6:** Result of zero-shot prediction on $D_{1}$.

Method	AG-ZSL			TF			FS-NET		
Attribute	Accuracy	Recall	Precision	Accuracy	Recall	Precision	Accuracy	Recall	Precision
chat_spike_fast_normal_TCP	0.8915	0.9036	0.7666	0.8701	0.8340	0.7913	0.8103	0.8213	0.7862
audio_steady_fast_normal_TCP	0.9177	0.9253	0.9155	0.8428	0.5726	0.6891	0.8603	0.6706	0.7032
file_burst_fast_normal_UDP	0.8616	0.8213	0.8389	0.8769	0.7913	0.8205	0.7483	0.6433	0.7699
audio_steady_slow_normal_SSH	0.8841	0.8387	0.8292	0.8723	0.8920	0.7995	0.8406	0.6856	0.6321
audio_steady_slow_normal_TCP	0.9113	0.8862	0.8063	0.8491	0.6716	0.7042	0.8527	0.7666	0.6967
audio_burst_slow_normal_TCP	0.9201	0.8901	0.8865	0.8482	0.7426	0.6712	0.7633	0.8516	0.7453
chat_steady_slow_normal_TCP	0.8712	0.8122	0.8977	0.8735	0.8083	0.8433	0.7630	0.7723	0.7906
audio_spike_fast_normal_UDP	0.7522	0.6130	0.7190	0.7302	0.5233	0.6234	0.7105	0.5150	0.6358
chat_spike_slow_normal_UDP	0.8083	0.7434	0.7540	0.7141	0.6215	0.6523	0.6488	0.6276	0.6520
audio_burst_fast_normal_TCP	0.7786	0.7556	0.7577	0.7212	0.7113	0.7417	0.6581	0.7066	0.7359
vedio_burst_slow_normal_UDP	0.7570	0.7101	0.7246	0.7363	0.7234	0.7281	0.6529	0.7313	0.7160
vedio_spike_slow_normal_TCP	0.7370	0.7072	0.716	0.7363	0.6063	0.6212	0.7226	0.5896	0.5568
Summary (Unseen classes)	0.7702	0.7290	0.7380	0.7269	0.6656	0.6858	0.6706	0.6637	0.6651
Summary (All classes)	0.8408	0.8005	0.8010	0.8059	0.7081	0.7238	0.7526	0.698	0.7017

**Table 7:** Complexity comparison.

Methods	Paramters	Training Time	Interfence Time	Overall Time	Performance (D1)					
					Fine-Grained Classification			Zero-Shot Prediction		
					Accuracy	Recall	Precision	Accuracy	Recall	Precision
AG-ZSL	62 MB	**1**	**1**	2	0.9571	0.9613	0.9578	0.8408	0.8005	0.8010
TF	6 MB	0.93	0.95	1.88	0.9171	0.9257	0.9182	0.8059	0.7081	0.7238
FS-Net	15 MB	0.84	0.98	1.82	0.9133	0.9254	0.9168	0.7526	0.6980	0.7017

## Data Availability

The source code of this paper can be obtained by sending an email to luzikui@bupt.edu.cn or chang030115@126.com.

## Appendix A. Attribute Label

The attribute labels in $D_{1}$ and $D_{2}$ are shown in the Table A1, where the bold labels represent the unknown categories in $Exp_{2}$ (which are known in $Exp_{1}$).

**Table A1:** The attribute labels in $D_{1}$ and $D_{2}$.

Scenario					
D1		D1			
No.	Attribute Label	No.	Attribute Label	No.	Attribute Label
1	chat_spike_fast_normal_TCP	1	injection_steady_fast_malware_DNS	21	polling_steady_slow_malware_DNS
2	audio_steady_fast_normal_TCP	2	injection_steady_slow_malware_HTTP	22	injection_spike_fast_malware_TCP
3	file_burst_fast_normal_UDP	3	control_burst_fast_malware_TCP	23	polling_burst_slow_malware_TCP
4	audio_steady_slow_normal_SSH	4	injection_steady_slow_malware_UDP	24	control_spike_fast_malware_UDP
5	audio_steady_slow_normal_TCP	5	control_steady_slow_malware_UDP	25	polling_spike_slow_malware_HTTP
6	audio_burst_slow_normal_TCP	6	polling_burst_fast_malware_TCP	26	polling_spike_slow_malware_SSH
7	chat_steady_slow_normal_TCP	7	polling_spike_fast_malware_HTTP	27	injection_burst_slow_malware_TCP
8	audio_spike_fast_normal_UDP	8	injection_burst_fast_malware_TCP	28	polling_steady_fast_malware_SSH
9	file_spike_fast_normal_TCP	9	polling_burst_fast_malware_HTTP	29	polling_burst_fast_malware_UDP
10	email_spike_fast_normal_UDP	10	injection_steady_fast_malware_TCP	30	injection_steady_slow_malware_FTP
11	chat_spike_fast_normal_HTTP	11	polling_burst_slow_malware_UDP	**31**	control_burst_slow_malware_HTTP
12	video_spike_slow_normal_HTTP	12	polling_steady_slow_malware_FTP	**32**	polling_burst_slow_malware_HTTP
13	audio_steady_slow_normal_UDP	13	injection_steady_fast_malware_HTTP	**33**	polling_burst_slow_malware_IMAP
14	email_burst_fast_normal_UDP	14	polling_burst_fast_malware_POP3	**34**	polling_steady_fast_malware_HTTP
15	audio_burst_fast_normal_UDP	15	control_spike_slow_malware_UDP	**35**	injection_burst_slow_malware_UDP
16	file_burst_fast_normal_UDP	16	control_spike_fast_malware_TCP	**36**	polling_spike_fast_malware_TCP
**17**	chat_spike_slow_normal_UDP	17	control_spike_slow_malware_HTTP		
**18**	audio_burst_fast_normal_TCP	18	polling_steady_slow_malware_UDP		
**19**	vedio_burst_slow_normal_UDP	19	polling_spike_fast_malware_POP3		
**20**	vedio_spike_slow_normal_TCP	20	polling_steady_slow_malware_NTP		
